# Adjacent Cell Marker Lateral Spillover Compensation and Reinforcement for Multiplexed Images

**DOI:** 10.3389/fimmu.2021.652631

**Published:** 2021-07-05

**Authors:** Yunhao Bai, Bokai Zhu, Xavier Rovira-Clave, Han Chen, Maxim Markovic, Chi Ngai Chan, Tung-Hung Su, David R. McIlwain, Jacob D. Estes, Leeat Keren, Garry P. Nolan, Sizun Jiang

**Affiliations:** ^1^ Department of Pathology, Stanford University, Stanford, CA, United States; ^2^ Department of Chemistry, Stanford University, Stanford, CA, United States; ^3^ Department of Microbiology and Immunology, Stanford University, Stanford, CA, United States; ^4^ Vaccine and Gene Therapy Institute and Oregon National Primate Research Center, Oregon Health & Science University, Beaverton, OR, United States; ^5^ Department of Internal Medicine, National Taiwan University Hospital, Taipei, Taiwan; ^6^ Department of Molecular Cell Biology, Weizmann Institute of Science, Rehovot, Israel; ^7^ Center for Virology and Vaccine Research, Beth Israel Deaconess Medical Center, Harvard Medical School, Boston, MA, United States

**Keywords:** multiplexed tissue imaging, spatial proteomics, signal spillover, image correction, single-cell biology, cell annotation

## Abstract

Multiplex imaging technologies are now routinely capable of measuring more than 40 antibody-labeled parameters in single cells. However, lateral spillage of signals in densely packed tissues presents an obstacle to the assignment of high-dimensional spatial features to individual cells for accurate cell-type annotation. We devised a method to correct for lateral spillage of cell surface markers between adjacent cells termed REinforcement Dynamic Spillover EliminAtion (REDSEA). The use of REDSEA decreased contaminating signals from neighboring cells. It improved the recovery of marker signals across both isotopic (i.e., Multiplexed Ion Beam Imaging) and immunofluorescent (i.e., Cyclic Immunofluorescence) multiplexed images resulting in a marked improvement in cell-type classification.

## Introduction

High-dimensional tissue imaging approaches such as CODetection by indEXing (CODEX), Multiplexed Ion Beam Imaging (MIBI), Cyclic Immunofluorescence (CyCIF), and imaging mass cytometry have contributed to our understanding of tissue biology and microenvironmental changes in disease (1–7). These methods, which are based on either fluorescence or isotope detection, retain the tissue context of single cells while enabling deep phenotyping capabilities (8). The accuracy of the phenotypic assignment of individual cells relies on several factors including marker specificity, instrument sensitivity, and segmentation accuracy. A significant amount of signal spillover can occur between segmented neighboring cells, particularly for signals due to robustly stained surface markers in regions of tissues densely packed with cells (i.e. lymph nodes, spleen, tumors, etc.). This lateral marker spillover differs from that due to overlapping excitation/emission spectra of fluorophores and from isotopic contamination and oxidation states (9, 10). Although the imperfect nature of cell segmentation contributes to this spillover, this phenomenon is observed even in well-segmented cells due to proximity and the interleaving of cell membranes of adjacent cells (3).

The confounding effects of signal spillover are usually identifiable by the false positive presence of markers on a cell that are generally considered mutually exclusive. Examples of mutually exclusive markers are CD3 and CD20, which are expressed on T and B cells, respectively, and CD4 and CD8, which are usually only simultaneously expressed on maturing T cells in the thymus (11). In mouse spleens imaged using CODEX, CD4/CD8 double positivity as high as 10% were observed seen due to spillover effects (3). Although the extent of these artifactual signals varies from platform to platform and depends on the markers stained and the tissue preparation methods, the effects complicate the identification of cell types, whether using manual gating methods or unsupervised algorithms. Considering that a large number of antibody tags (>40) are now routinely imaged in multiplexed studies (4, 7, 12–14), cumulative pairwise spillovers can have detrimental effects on data quality.

Here, we present a spillover compensation algorithm, REinforcement Dynamic Spillover EliminAtion (REDSEA), that is robustly applicable to several imaging modalities. We reasoned that spillover from segmented adjacent cells could be corrected based on the proportion of the shared boundary between cells, focusing only on pixels near the periphery between adjacent cells. Importantly, REDSEA correctly reassigns spillover signal, even low-abundance ones, to the cell of origin.

An ideal correction algorithm should be unsupervised and require no *a priori* knowledge of how the markers are distributed with the inputs being the segmentation map and the single-channel TIFF files (or equivalent) from the multiplexed images. The output is an FCS file containing the extracted per-channel quantification of the single-cell data in both the original and compensated formats which can then be used in various clustering algorithms to “recolor” the original image with cell types or other derived features such as cell neighborhood participation or cell activation state. The modular nature of such software should allow the use of pre-processing methods, and the algorithmic output that can be used directly in various downstream analysis approaches. REDSEA is thereby our contribution towards such a goal. The software implemented here is freely available from https://github.com/nolanlab/REDSEA. We applied REDSEA to datasets from two types of highly multiplexed tissue imaging approaches: mass spectrometry based MIBI and immunofluorescence-based CyCIF. Application of REDSEA resulted in a marked reduction in double positivity for known mutually exclusive markers (e.g., CD3 and CD20; CD4 and CD8a) and enrichment of cell lineage markers in expected cell types. The signals from low abundance tags remained robust after correction, indicating that the signal was appropriately reassigned to cells of origin. We finally demonstrated the use of REDSEA for sensitive and accurate stratification of cell types using unsupervised methods. The REDSEA method will improve the sensitivity and accuracy of cell-type annotation of segmented cells in highly multiplexed imaging studies using both fluorescence and isotope-based strategies.

## Results

### REDSEA Reduces Lateral Spillover and Boosts Marker-Specific Signals in Multiplexed Ion Beam Images

**Figure 1:**
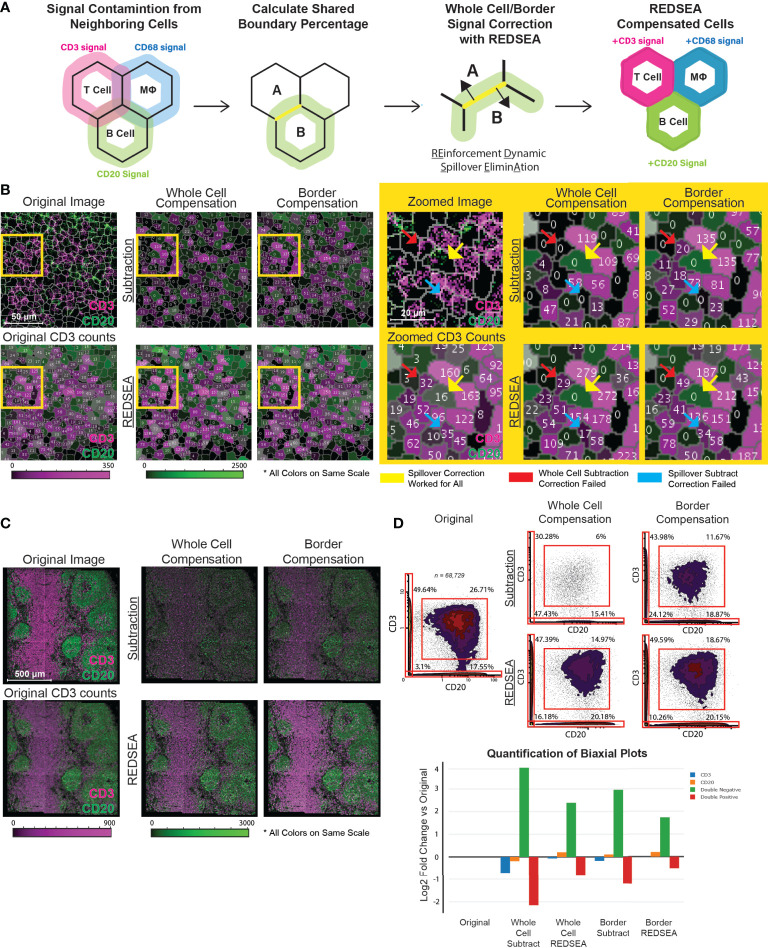
REDSEA Corrects Signal Spillover between Adjacent Cells. **(A)** A schematic representing the workflow and principles of REDSEA compensation. The spillover signal from neighboring cells is dynamically eliminated based on the fraction of the shared boundary between neighboring cells and the signal intensity. **(B)** Left: A representative 150 µm x 150 µm MIBI image of a rhesus macaque lymph node. Two mutually exclusive markers are shown (CD3, magenta; CD20, green), and the numerical counts of CD3 are indicated in each segmented cell before and after images were subjected to spillover subtraction using the following four methods: 1) spillover subtraction on the whole cell, 2) spillover subtraction on only the border region, 3) REDSEA compensation on the whole cell and 4) REDSEA only on the border regions. The CD3 and CD20 counts per cell are colored on the same scale for the segmented cells across compensation settings. Right: Zoomed images of the yellow boxed regions on the left. The yellow arrows indicate representative cells for which CD3 spillover was successfully corrected by all the four methods; red arrows indicate successful correction by all but the whole cell subtraction method, and blue arrows indicated successful correction only by REDSEA-based and not the other compensation methods. **(C)** A representative 1200 µm x 1200 µm MIBI image of a rhesus macaque lymph node subjected to spillover corrections as indicated above in **(B)**. **(D)** Top: Biaxial plots of marker intensities of 68,739 single cells from segmented MIBI images of rhesus macaque lymph nodes. The percentage of single-positive (top left and bottom right quadrants), double-positive (top right quadrant), and double-negative (bottom left quadrant) cells are shown for each compensation method. Bottom: A log2 fold change plot (compensated over original non-compensated) for single-positive, double-positive, and double-negative gated populations.

Cell segmentation is commonly used to assign and extract features from images to individual cells, allowing downstream analysis to be performed while retaining the spatial contexts of features as we previously observed (15–18). Segmentation algorithms can vary in performance, and we generally find DeepCell performs well in MIBI images compared to other alternatives (**Figure S1A**) (4, 18). Even in well segmented cells, however, lateral signal spillover from surface markers into adjacent cells is common (**Figures 1A, B** and **S1B**).

We observed that antibody-dependent signals in cell staining and imaging are generally evenly distributed around a cell for most markers in common use. Further, the quantity of the spillover signal from a single channel into a neighboring cell is directly associated with the strength of that channel signal in the originating cell. Thus, we reasoned that the subtraction of this artifactual signal as a fraction of the signal in the originating cell would allow the correct assignment of signals to individual cells. We also hypothesized that a border-based subtraction, instead of whole-cell subtraction, would better model the nature of the spillover. Finally, we postulated that a reinforcement methodology would allow the attribution of missing signals back to their originating cell.

Reasonable unsupervised performance of REDSEA requires at least two underlying assumptions: 1) The signals from the marker to be corrected are generally uniformly distributed around the boundary of the originating cell, and 2) the signals from the tag to be corrected is higher inside the originating cell compared to the spillover signals outside. Thus, the REDSEA approach first calculates the percentage boundary overlap of adjacent cells and extracts signals with respect to this boundary (**Figure 1A**). To allow flexibility for application to any imaging modality and resolution, the determination of this boundary can be user defined by the distance from the boundary (**Figure S1C**). Next, the signals are compensated in the overlapping boundary region or the whole cell for each pair of adjacent cells (**Figures 1A** and **S1D**).

We first validated our approach using multiplexed MIBI images of rhesus macaque lymph nodes. MIBI data were processed as previously described (14), and segmentation performed *via* DeepCell by training the segmentation model on manually segmented cells based on dsDNA (**Figure S1E**). The four spillover correction strategies were implemented on the features extracted from single-cells after nuclear segmentation (4). The four correction strategies were 1) spillover subtraction on the whole cell, 2) spillover subtraction on only the border region, 3) REDSEA compensation on the whole cell, and 4) REDSEA only on the border regions. We first tested the algorithms on two markers with expression known to be mutually exclusive, CD3 and CD20. If the compensation works well, one would expect a reduction of single-cells with both CD3 and CD20 signals, while retaining a comparable number of CD3^+^ and CD20^+^ single-positive cells. Although all the strategies effectively removed spillover signals (**Figures 1B–D**), border-based subtraction methods better modeled the nature of the spillover with less diminution of channel signals than did whole-cell subtraction methods while retaining the ability to correct for spillover (**Figures 1B, D**). Specifically, REDSEA based compensation methods retained the robustness of the CD3 and CD20 signal counts per cell, similar to the original non-compensated levels (**Figure 1B**, left and **Figure 1C**). While all four methods were able to correct the spillover in some cells (exemplified in **Figure 1B**, right, yellow arrows), the subtraction-only based correction methods failed in various circumstances (illustrated in **Figure 1B**, right, red and blue arrows).

REDSEA, which has both subtraction and reinforcement components, maintained signal intensity after correction to a greater extent than the spillover subtraction algorithm (**Figure 1B**, see right panels for magnified image). Quantification of single- or double-positive cells in the pre- and post correction strategies demonstrated that the REDSEA method outperformed the subtraction strategy (**Figure 1D**). The initial double-positive cell rate of 26.71% (total: 68,729 cells) was reduced after compensation to 6% for whole cell compensation, 11.67% for border compensation, 14.97% for whole cell REDSEA and 18.67% for border REDSEA (**Figure 1D**, right panel, upper right quadrant). Although the subtraction-only correction methods resulted in a greater decrease in double-positive cells, this strategy also resulted in a marked increase in double-negative cells from 3.1% initially to 47.43% for whole cell compensation, 24.12% for border compensation (**Figure 1D**, right panel, lower left quadrant). This substantial loss of marker signal was rescued by applying the REDSEA algorithm to a lower 16.18% for whole cell REDSEA and 10.26% for border REDSEA (**Figure 1D**, right panel, lower left quadrant). Proportions of both the CD3^+^ and CD20^+^ single-positive cells also diminished after subtraction-only corrections but was maintained or increased by REDSEA from the initial 49.64/17.55% (CD3^+^/CD20^+^) to 30.28/15.41% for whole cell compensation, 43.98/18.87% for border compensation, 47.39/20.18% for whole cell REDSEA and 49.59/20.15% for border REDSEA (**Figure 1D**, right panel, upper left and lower right quadrants).

Inspection of a number of double-positive cells not corrected by REDSEA indicates REDSEA-independent factors, such as segmentation imperfections or physically overlapping cells (**Figures S1F, G**). In subsequent analyses, unless otherwise noted, REDSEA corrections were performed with the REDSEA border compensation method to maximize spillover compensation while minimizing signal loss.

### REDSEA Reduces Non-Specific Spillover Signals and Boosts Marker Specific Signals

**Figure 2:**
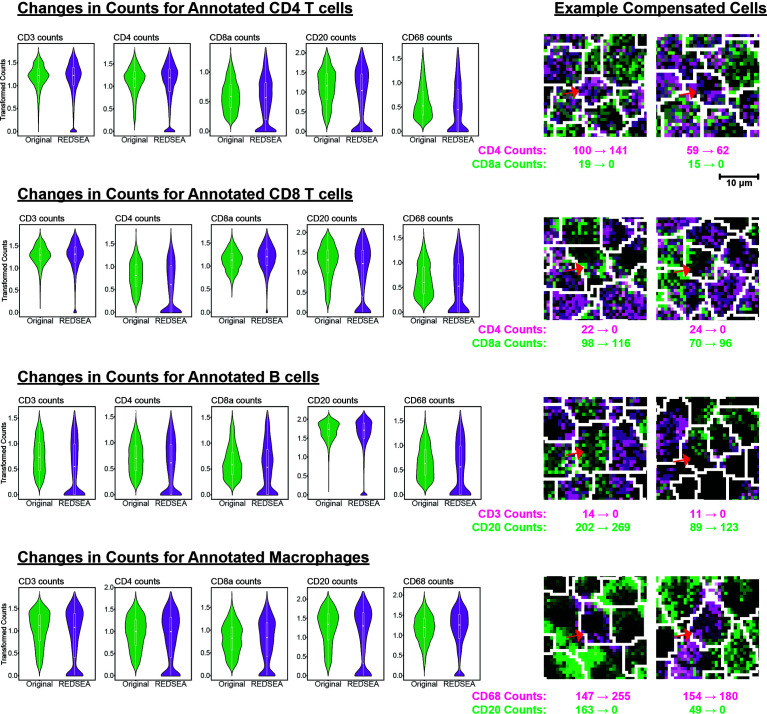
REDSEA Reduces Non-Specific Spillover Signals. Left: Arcsine and square root transformed counts per cell for CD3, CD4, CD8a, CD20, and CD68 were plotted before and after REDSEA border compensation for each of the cell types identified. Right: Representative images of each cell type with marker counts before and after REDSEA compensation.

We next assessed how REDSEA reduced and reinforced cell-type-specific counts for various lineage specific cell surface markers. By plotting the original or REDSEA compensated surface marker signals per cell for each group of cell types identified using unsupervised means (see *Materials & Methods*), we hypothesized that lineage-specific markers would increase while nonspecific markers should decrease. Indeed, we observed an increase or retention in the signal after REDSEA for CD3 and CD4 T cell markers in CD4 T cells (**Figure 2**, row 1), CD3 and CD8 T cell markers in CD8 T cells (**Figure 2**, row 2), B cell marker CD20 in B cells (**Figure 2**, row 3), CD68 and CD163 macrophage markers in macrophages (**Figure 2**, row 4 and **Figure S2**). Together, this is indicative that the reinforcement aspect of REDSEA operates correctly to boost lineage specific marker signals in respective cell-types.

Conversely, we observed a decrease in non-lineage specific markers for CD4 T cells, CD8 T cells, B cells, and macrophages (**Figures 2** and **S2**). These results confirm that non-specific marker signals, such as lateral membrane spillover from adjacent cells, were successfully reduced or eliminated by REDSEA. Representative examples of cells with increased cell-type specific signals and reduced cell-type non-specific signals are highlighted (**Figures 2** and **S2**, right).

**Figure 3:**
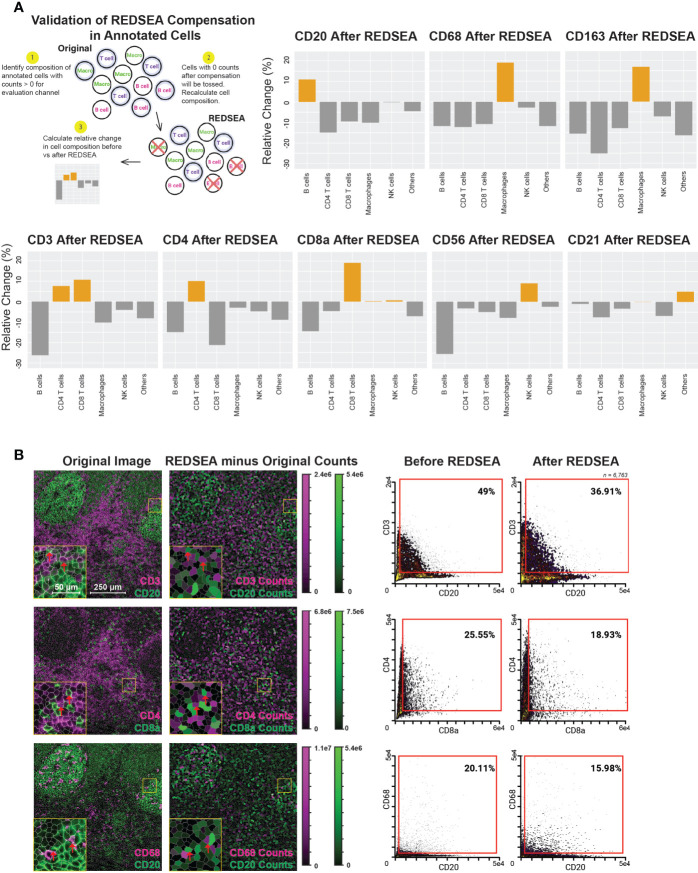
REDSEA Enriches for Cell-type-specific Signals and is Platform Agnostic. **(A)** Schematic of the workflow for calculation of enrichment and depletion of various cell types for each channel before and after REDSEA correction. Cells with no counts in the channel of interest after REDSEA correction were discarded, and the percentage composition of each cell type remaining was calculated. The relative change is the difference in percentage composition of each cell type before and after REDSEA correction. **(B)** Left: A representative 900 µm x 900 µm CyCIF image of a human tonsil. Three pairs of mutually exclusive markers are shown: CD3 (magenta) and CD20 (green); CD4 (magenta) and CD8a (green); and CD68 (magenta) and CD20 (green). The differences in percentage compositions between the REDSEA image and the original in counts of both markers per segmented cell are shown on a visual scale. Right: Biaxial plots of marker signals from each of the 6,295 single cells extracted from the segmented CyCIF images. The percentage composition of double-positive (top right quadrant) cells is shown for each compensation method.

We then devised a strategy to calculate whether previously identified cell-types were enriched in a marker-specific manner after REDSEA: 1) Only cells containing positive counts for a specific marker of interest (e.g., CD20) were considered initially, and the percentage composition of each cell type calculated. 2) Cells with 0 counts for the marker of interest after REDSEA compensation are dropped, and the new percentage composition of each cell type recalculated. 3) The relative change in percentage composition (after/before) of each cell type was then determined and plotted (**Figure 3A**, row 1 left).

We observed a post-REDSEA enrichment of B cells after CD20 cleanup and macrophages after CD68 or CD163 cleanup (**Figure 3A**, top row). Similarly, CD4 and CD8 T cells were enriched after cleanup of CD4 and CD8a respective, and both improved upon pan-T cell marker CD3 cleanup (**Figure 3A**, bottom row). CD56 yielded an enriched composition of NK cells, while CD21 cleanup appears to enrich cell types not annotated as part of this study. These CD21 enriched, unannotated cells are likely follicular dendritic cells (19). This quantification of the enrichment for cell types associated with a particular surface marker demonstrated the effectiveness of this methodology of spillover clean up.

### REDSEA Corrects Aberrant Spillover Signal in Immunofluorescence Multiplexed Images

To ensure the platform-agnostic nature of this method, we applied REDSEA to publicly available images generated using CyCIF, an immunofluorescence-based multiplexed imaging modality (6, 20). For this study, we focused on CyCIF images generated from human tonsils, to demonstrate the robustness of REDSEA for tightly packed lymphoid tissues. To improve original cell segmentation provided, we retrained an established convolutional neural network to better identify and segment single cells (**Figure S3A**). We next extracted single-cell features before and after REDSEA correction and calculated the difference in signal intensity for mutually exclusive markers CD3 & CD20, CD4 & CD8a, and CD68 & CD20 due to REDSEA correction (**Figure 3B**, left). We observed an increase in CD20 counts due to REDSEA in B cell follicles, and an increase in CD3, CD4, and CD8a counts in T cell zones (**Figure 3B** left and **Figure S3B**, rows 1-2, red arrows). An increase in CD68 positive cells was also distributed throughout the B cell follicle and T cell zones, indicative of robust compensation of macrophages (**Figure 3B** left and **Figure S3B**, row 3, red arrows). Biaxial quantification of these single-cell signals before and after REDSEA corroborated with the spatial representation, showing a decrease of double-positive cells for these of mutually exclusive markers (**Figure 3B**, right). These results demonstrated that REDSEA corrects aberrant spillovers in mass spectrometry-based and fluorescence-based multiplexed imaging modalities.

### Unsupervised Cell-Type Annotation Is Improved After REDSEA Correction

To assess the material benefit of REDSEA to empirical data, we performed unsupervised meta clustering, and cell-type identification (21, 22) of 1836 cells from a single MIBI field-of-view acquired on a rhesus macaque lymph node stained with 11 markers (see *Materials & Methods*), using the uncompensated original markers values and compensated values from each of the four correction methods described above (whole-cell subtraction or REDSEA, border subtraction or REDSEA).

From our experience with MIBI data, a few iterative rounds of unsupervised classification are generally sufficient to identify most cell types present. This is generally due to variable expression of the markers of interest, or confounding factors such as lateral marker spillover. We postulated that a proper way to benchmark the benefits of lateral spillover on unsupervised cell-type annotation would be to perform a single round of classification, at a fixed number of pre-set clusters. Comparisons to a manually curated set of cell-type annotations would then allow quantification of sensitivity and accuracy between the original state and four methods.

**Figure 4:**
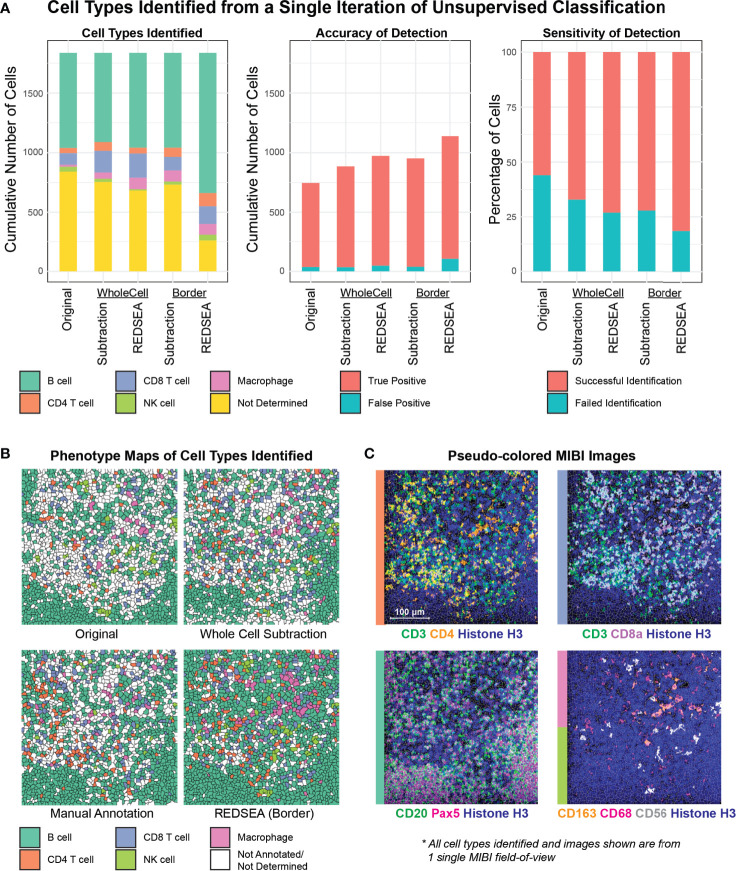
REDSEA Improves Cell-type Annotation of MIBI images. **(A)** Single-cell marker values were determined from a single MIBI field of view of a rhesus macaque lymph node (400 µm x 400 µm) with no corrections (Original) or 1) spillover subtraction on the whole cell, 2) REDSEA compensation on the whole cell, 3) spillover subtraction on only the border region and 4) REDSEA only on the border regions. A single iteration of unsupervised cell type classification was performed with identical parameters on extracted single cell marker values under each of the conditions. The quantification of cell types identified in terms of fold change (left) and cumulative number (right) are represented here. Not determined (yellow) denotes cell types that could not be confidently assigned to clusters identified during classification. **(B)** Spatial positions of cell types identified under each condition are represented as phenotype maps. Phenotype maps for cell types identified without correction (Original), with whole cell-based spillover subtraction (Whole Cell Subtraction), with consensus-based manual annotation from three independent individuals (Manual Annotation) and with border-based REDSEA [REDSEA (Border)] are shown. **(C)** Pseudo-colored MIBI images containing various combinations of cell-type-specific markers from the same field of view.

Of the five conditions above (original and four compensation algorithms), 30 clusters were identified *via* the FlowSOM algorithm (22) using CD3, CD4, CD8a, CD20, CD21, Pax-5, CD56, CD163, and CD68, before subjection to Marker Enrichment Modeling (21) to classify CD4 T cells, CD8 T cells, B cells, NK cells and macrophages in an unsupervised fashion. The effects of lateral compensation were immediately apparent: marker expressions within the cell clusters identified in the original population were more ambiguous than the distinctive compensated plots (**Figure S4A**). Indeed, REDSEA border correction resulted in a notable decrease in the number of cells requiring further iterative clustering and an increase in cells that were confidently annotated (**Figure 4A** left and **Figure S4B**). For cells that could not be assigned a cell type from this single-round of classification, we identified 841 in the original condition, 754 after whole cell subtraction, 682 after whole cell REDSEA, 732 after border subtraction and 259 after border REDSEA compensation. This indicates that lateral correction using border REDSEA can reduce non-specific signals which confound the unsupervised classification process.

We then evaluated the accuracy and sensitivity of cell-type classification using manual cell-type annotations from three independent individuals (ground truth). We defined accuracy as the portion of unsupervised cell annotations that was correct compared to the ground truth. 710 cells were correctly identified in the original condition, 851 after whole cell subtraction, 926 after whole cell REDSEA, 914 after border subtraction, and 1033 after border REDSEA compensation (**Figure 4A** middle). The percentage accuracy for each cell type were also comparable across the methods (**Figure S4C**, left), with more variability for NK cells due to the low numbers present.

We next defined sensitivity as the portion of cell types annotated in the ground truth that was identified by the unsupervised clustering under each condition. 56.0% of cells were successfully identified in the original condition, 67.1% after whole cell subtraction, 73.0% after whole cell REDSEA, 72.1% after border subtraction, and 81.5% after border REDSEA compensation (**Figure 4A** right). The percentage sensitivity for each cell type was also generally increased for REDSEA corrected cells (**Figure S4C**, right).

The median marker expression for cell types identified in each case also reflects a reduction of non-specific signals, and enrichment of the appropriate cell-type-specific signals, such as CD3 for T cells and CD68 and CD163 for macrophages (**Figure S4D**). To visually confirm our annotations, annotated cells under each condition were plotted by their phenotypes spatially, showing an expected cell-type distribution when compared to ground truth (**Figures 4B** and **S4E**). The lower sensitivity of the original and subtraction methods was apparent from the large patches of white, unannotated cells in the phenotype maps (**Figures 4B** and **S4E**). We also plotted pseudo-colored MIBI images of the lineage-specific markers used for the unsupervised annotation (**Figure 4C**).

These results indicate that REDSEA will improve unsupervised cell-type classification real-world performance, speeding up a process that can be confounded otherwise by signal spillover.

## Discussion

Increases in the number of markers measured on the same section of tissue, coupled with the use of unsupervised methods to identify cell populations, have allowed breakthroughs in our understanding of tumor microenvironments and cellular interactions (1–7, 14). However, the spillover of marker signals between segmented cells can confound unsupervised identification methods leading to misinterpretation of the data, and spillover is particularly difficult to isolate in the presence of 40 or more markers. To resolve this in a systematic and unsupervised way, we have developed the REDSEA algorithm to correct for marker spillover between segmented cells in multiplexed images.

Here, we validated REDSEA by analysis of mass spectrometry and immunofluorescence imaging experiments and demonstrated the platform-agnostic capabilities of our method. Although the focused nature of a primary oxygen beam on the MIBI results in fewer spillover issues than observed with immunofluorescence lasers (1, 5), the number of cells positive for both CD3 and CD20, which are not expressed on the same cell, show that spillover is a challenge for both modalities of imaging on packed lymphoid tissues (MIBI and CyCIF).

The current iteration of REDSEA has some limitations: First, it does not perform 3D corrections. Second, it is unable to correct for situations where multiple cells physically overlap. Third, REDSEA can only correct for lateral marker spillover, and not signal spillovers due to autofluorescence or imaging artefacts such as overlapping excitation/emission spectra or isotopic contamination. Fourth, its performance is dependent upon proper segmentation of cells, possible now with recently improved methods (17, 18, 23). Despite this, we show that it mitigates spillover issues in multi-parametric spatial data analysis (24) and enables greater cell recovery from imaging datasets.

In conclusion, REDSEA effectively corrects for channel spillover between adjacent cells in multiplexed imaging data in an unsupervised fashion that requires only the raw per channel TIFF images and a segmentation layer. Thus, REDSEA can be used to minimize confounding effects, reduce misinterpretation of the imaging data, and has practical applications for improving unsupervised cell type identification with current state-of-the-art methods.

## Materials and Methods

### Antibodies

Antibodies were conjugated to metal polymers using the Maxpar X8 Multimetal Labeling Kit (201300, Fluidigm) as per manufacturer protocols. The antibodies used, their respective clones and channels are listed in **Table S1**.

### Gold Slide Preparation

Gold slides were prepared as previously described (14). Briefly, Superfrost Plus glass slides (Thermo Fisher Scientific, #12-552-3) were soaked in dish detergent, rinsed with distilled water, and dried with airflow to remove water drops. The slides were first coated with 30 nm of Tantalum and then with 100 nm of gold at the Stanford Nano Shared Facility (SNSF).

### Animal Ethics Statement

FFPE tissues were obtained from SIV-infected and SIV-negative rhesus macaques (Macaca mulatta) of Indian origin that were housed at the Oregon National Primate Research Center (OR, USA) and at the National Institutes of Health (Bethesda, MD, USA) with the approval of the respective Institutional Animal Care and Use Committees. Animal experiments were conducted following guidelines set forth by the NIH and the Animal Welfare Act and in accordance with American Association for the Accreditation of Laboratory Animal Care (AAALAC) standards in AAALAC-accredited facilities.

### Vectabond Pre-treatment of Gold Slides

Gold slides were immersed in 100% acetone for 5 min and then incubated in a mixture of 2.5 ml Vectabond (Vector Labs, #SP1800) and 125 ml of 100% acetone in a glass beaker for 30 min. Slides were subsequently washed in 100% acetone for 30 s, then dipped in distilled water to remove any residues, air dried, and stored at room temperature.

### Staining

The tissue was sectioned onto gold slides at 4-µm thickness and stored in a vacuum chamber. Before staining, slides were baked for 1 h at 70°C and soaked in xylene for 3 x 10 min. Standard deparaffinization was performed thereafter (3 x xylene, 2 x 100% EtOH, 2 x 95% EtOH, 1 x 80% EtOH, 1 x 70% EtOH, 3 x ddH_2_O; 3 min each). Epitope retrieval was then performed at 97°C for 10 min at pH 9 (Dako Target Retrieval Solution, S236784-2) in a Lab Vision PT Module (Thermo Fisher Scientific).

Slides were cooled to 65°C in the PT Module and then removed for equilibration to room temperature. Tissue regions were marked using a PAP pen (Vector Labs). Slides were rinsed 2 x 5 min in MIBI Wash Buffer (1X TBS-T, 0.1% BSA). The slides were then blocked in MIBI Blocking Buffer (1X TBS-T, 2% donkey serum, 0.1% Triton X-100, 0.05% sodium azide) for 1 h. Finally, the antibody cocktail (antibodies in 1X TBS-T, 3% donkey serum, 0.05% sodium azide) was added and left at 4°C overnight.

The following day, slides were washed 3 x 5 min in MIBI Wash Buffer, before crosslinking in Fixation Buffer (2% glutaraldehyde, 4% PFA in 1X PBS) for 15 min. Slides were then rinsed once in 1X PBS. Quenching of crosslinkers was performed for 3 x 1 min in 100 mM Tris, pH 7.5. Slides were then dehydrated in increasing concentrations of EtOH (3 x ddH_2_O, 1 x 70% EtOH, 1 x 80% EtOH, 2 x 95% EtOH, 2 x 100% EtOH). Slides were kept in a vacuum desiccator until imaging on the MIBI-TOF (Ionpath Inc).

### MIBI-TOF Data Acquisition and Processing

Mass imaging was performed on a custom MIBI-TOF mass spectrometer equipped with a duo plasmatron ion source (Ionpath Inc, 5). All images in this study were acquired using the following parameters: • Pixel dwell time: 12 ms • Image size: 400 µm x 400 µm at 512 x 512 pixels • Probe size: 400 nm • Primary ion current: 3.5 nA as measured *via* a Faraday cup on the sample holder • Number of depths: 3 MIBI images were extracted and denoised using MIBIAnalysis tools (https://github.com/lkeren/MIBIAnalysis) as previously described (4). All three depths were aligned and summed for the purpose of this study.

### Image Segmentation

Cell segmentation was performed using the DeepCell convolutional neural network as previously described (4, 18). The training dataset consists of denoised MIBI images for Histone H3 and dsDNA that were cropped and randomly chosen for manual segmentation using a Wacom Tablet (Wacom Intuos Draw). A watershed algorithm was applied to the nucleus possibility map to segment the image into individual cells (4). The segmentation was performed with the parameters segmentThres = 0.01 and probNucThre = 0.35.

### Adjacency Compensation Methodology and Implementation

In the segmentation map, pixels belonging to individual cells were labeled with the same number as a unique cell identifier and separated with a one-pixel wide continuous boundary labeled with zeros. The first step of the REDSEA algorithm loops through all the boundary pixels and searches in a 3x3 grid for cell labels. The boundary between two adjacent cells is then calculated as a percentage of the perimeter of each of the two cells, respectively. A sparse matrix is then assembled with these values as the coefficient of pairwise compensation. In the second step, the algorithm loops through every pixel in each cell with a user-defined pixel number and structuring element (either sudoku or star, **Figure S1C**) in search of boundary pixels. We found that a pixel number of 2 and a star structuring element (which performs a search in the 12 surrounding pixels) worked well with for REDSEA on our MIBI images. Once cell boundary pixels were located and the counts collected along the cell boundary, the Subtracted Signal for channel *X* counts of cell *A* was determined using the following equation:

(1) $$Subtracted\;Signal=\sum_{K=1}^{n}\left(X_{K_{0}}^{b}\times \frac{b_{AK}}{P_{K}}\right)$$

where $X_{AK}^{b}$ denotes the boundary pixel counts for each cell *K* that shares a common boundary with cell *A* for *n* number of cells; *b_AK_*denotes the length of the shared boundary between cell *A* and cell *K*; and *P_K_* is the perimeter of cell *A*.

REDSEA then reinforces the subtracted signal back to the originating cell. In this case, the reinforced signal is the sum of all the boundary signals for all *n* number of cells *K* around cell *A*:

(**2**) $$Reinforced\;Signal=\sum_{K=1}^{n}\left(X_{A_{0}}^{b}\times \frac{b_{AK}}{P_{A}}\right)$$

Taking both the subtraction and reinforcement together, we obtain the equation:

(3) $$X_{A_{comp}}=X_{A_{0}}+\sum_{K=1}^{n}\left(X_{A_{0}}^{b}\times \frac{b_{AK}}{P_{A}}\right)-\sum_{K=1}^{n}\left(X_{K_{0}}^{b}\times \frac{b_{AK}}{P_{K}}\right)$$

(4) $$=X_{A_{0}}+\left(\sum_{K=1}^{n}b_{AK}\right)\times \frac{X_{A_{0}}^{b}}{P_{A}}-\sum_{K=1}^{n}\left(X_{K_{0}}^{b}\times \frac{b_{AK}}{P_{K}}\right)$$

(5) $$=X_{A_{0}}+X_{A_{0}}^{b}-\sum_{K=1}^{n}\left(X_{K_{0}}^{b}\times \frac{b_{AK}}{P_{K}}\right)$$

where *X_Acomp_*denotes the counts of channel *X* for cell *A* after REDSEA compensation. For simplicity, we assume that $\Sigma_{K=1}^{n}\;b_{AK}=P_{A}$. If *X_Acomp_* is negative, the final count is returned as zero. These equations are based on two underlying assumptions for a typical cell surrounded by neighboring cells, with mutual signal spillovers (**Figure S1D**, left): First, the spillover, generally due to a cell-surface marker, is uniformly distributed around the originating cell boundary. Second, in well-segmented cells, the signal of the marker is higher inside the segmentation than outside.

### Comparison of Marker Intensity Before and After REDSEA

To compare the signal intensity of each channel before and after REDSEA compensation, cells with counts > 0 for the channel of interest before REDSEA were selected. For visualization purposes, marker signals per cell were normalized by cell size (pixel number), arcsine transformed (cofactor = 1), and a final square root transformation. The distribution of transformed counts before and after REDSEA was plotted for each of the cell types annotated.

### Enrichment Calculation of Cell Types Before and After REDSEA

To evaluate the effect of cell type enrichment before and after REDSEA compensation, cells with counts > 0 for the channel of interest before REDSEA were selected, and the percentage of each cell type based on cell-type annotation described above was calculated; After compensation, cells with count > 0 were retained, and the new cell-type composition was calculated based on the remaining cells. The relative percentage enrichment of cell types was defined as:

(6) $$\frac{Percentage\;after-Percentage\;before}{Percentage\;before}\%$$

### Application of REDSEA to Immunofluorescent Images

The CyCIF human FFPE tonsil dataset used in this study was downloaded from Synapse (Tonsil-1 40x, https://www.synapse.org/#!Synapse:syn17796423) (20). The channels selected for REDSEA normalization were DAPI, CD3, CD4, CD8a, CD3, and CD68. For more accurate segmentation of the results compared to the provided segmentation map, a custom DeepCell neural network was trained based on the DAPI channel as described in the “Image Segmentation” section. A watershed algorithm was applied for whole-cell segmentation. The segmentation was performed with the parameters segmentThres = 0.05 and probNucThre = 0.05. The size of the structuring element was adjusted to 4 pixels to account for differences in the image sizes between MIBI and CyCIF data, and a star pattern (**Figure S1C**) was used to perform a search of 40 pixels around the border pixel to obtain consistent REDSEA performance.

### MIBI Image Analysis and Cell-Type Annotation

Features from single cells in segmented MIBI images were extracted under each compensation condition using the same segmentation map. Markers for each cell were normalized by their median dsDNA levels for each field of view and rescaled to a 0 - 1 range. Unsupervised classification of cell types was performed with FlowSOM (22) on the markers (CD3, CD4, CD8a, CD20, CD21, Pax-5, CD68, CD163 and CD56) for 30 clusters, and cell types were identified from each cluster with marker enrichment modeling (21).

### Cell-Type Sensitivity and Accuracy Calculations

Cell-type sensitivity and accuracy were determined as follows:

(7) $$Sensitivity=\frac{Number\;of\;cell‐type\;A\;correctly\;called\;by\;algorithm}{Total\;Number\;of\;cell‐type\;A\;in\;ground\;truth}\%$$

(8) $$Accuracy=\frac{Number\;of\;cell‐type\;A\;correctly\;called\;by\;algorithm}{Total\;Number\;of\;cell‐type\;A\;called\;by\;algorithm}\%$$

### Visualize Illustration and Plotting of Data

All plots associated with this manuscript, with the exception of biaxial plots, were generated using ggplot2 (25).

### Biaxial Quantification of Single-Cell Data

All biaxial plots and quantification were generated using CellEngine at https://immuneatlas.org/(Primity Bio).

### Software

Executable MATLAB scripts of the adjacency compensation method described here, as well as detailed instructions, are available at https://github.com/nolanlab/REDSEA.

## Data Availability Statement

The code and data generated in this study is available at https://github.com/nolanlab/REDSEA. The names of the public repositories and their relevant accession links are detailed in the *Materials and Methods* section.

## Ethics Statement

The animal study was reviewed and approved by FFPE tissues were obtained from SIV-infected and SIV-negative rhesus macaques (Macaca mulatta) of Indian origin that were housed at the Oregon National Primate Research Center (OR, USA) and at the National Institutes of Health (Bethesda, MD, USA) with the approval of the respective Institutional Animal Care and Use Committees. Animal experiments were conducted following guidelines set forth by the NIH and the Animal Welfare Act and in accordance with American Association for the Accreditation of Laboratory Animal Care (AAALAC) standards in AAALAC-accredited facilities.

## Author Contributions

Conception and design: SJ, YB, BZ. Sample preparation and collection of data: SJ, YB, BZ, and XR-C. Algorithm implementation: YB, BZ, and SJ. Analysis and interpretation of data: YB, BZ, SJ. Contribution of reagents and tools: HC, MM, CC, T-HS, DM, JE, and LK. Supervision: SJ and GN. Manuscript preparation: SJ, YB, BZ, and GN. The co-first authorship order was determined *via* the best of three rounds in Super Smash Bros. Both YB and BZ contributed equally and have the right to list their name first in their CV. All authors contributed to the article and approved the submitted version. 

## Funding

SJ is supported by the Leukemia & Lymphoma Society Career Development Program. BZ is supported by a Stanford Graduate Fellowship. This work was funded in part by grants from the National Cancer Institute, National Institutes of Health, Task Order No. HHSN261100039 under Contract No. HHSN261201500003I (GN) and U2CCA233195 (GN), the National Institutes of Health R01AI149672 (GN and JE), the Bill & Melinda Gates Foundation INV-002704 (GN and JE), OPP1113682 (GN), and COVID-19 Pilot Award (SJ, DM, GN), the Fast Grant Funding for COVID-19 Science (GN), the Food and Drug Administration HHSF223201610018C (GN), the Parker Institute for Cancer Immunotherapy (GN), and the Rachford and Carlota A. Harris Endowed Professorship (GN). This article reflects the views of the authors and should not be construed as representing the views or policies of the FDA, NIH, BMGF or other institutions who provided funding.

## Conflict of Interest

GN is a co-founder and has a personal financial interest in the company IonPath, which manufactures the instrument used in this manuscript.

The remaining authors declare that the research was conducted in the absence of any commercial or financial relationships that could be construed as a potential conflict of interest.
