# Supplementary material for: Adjacent Cell Marker Lateral Spillover Compensation and Reinforcement for Multiplexed Images
Source: Front Immunol. 2021 Jul 5;12:652631. doi: 10.3389/fimmu.2021.652631 (PMC8289709; doi:10.3389/fimmu.2021.652631)
Supplement: Supplementary file 1 [file DataSheet_1.pdf]

Supplementary Figures

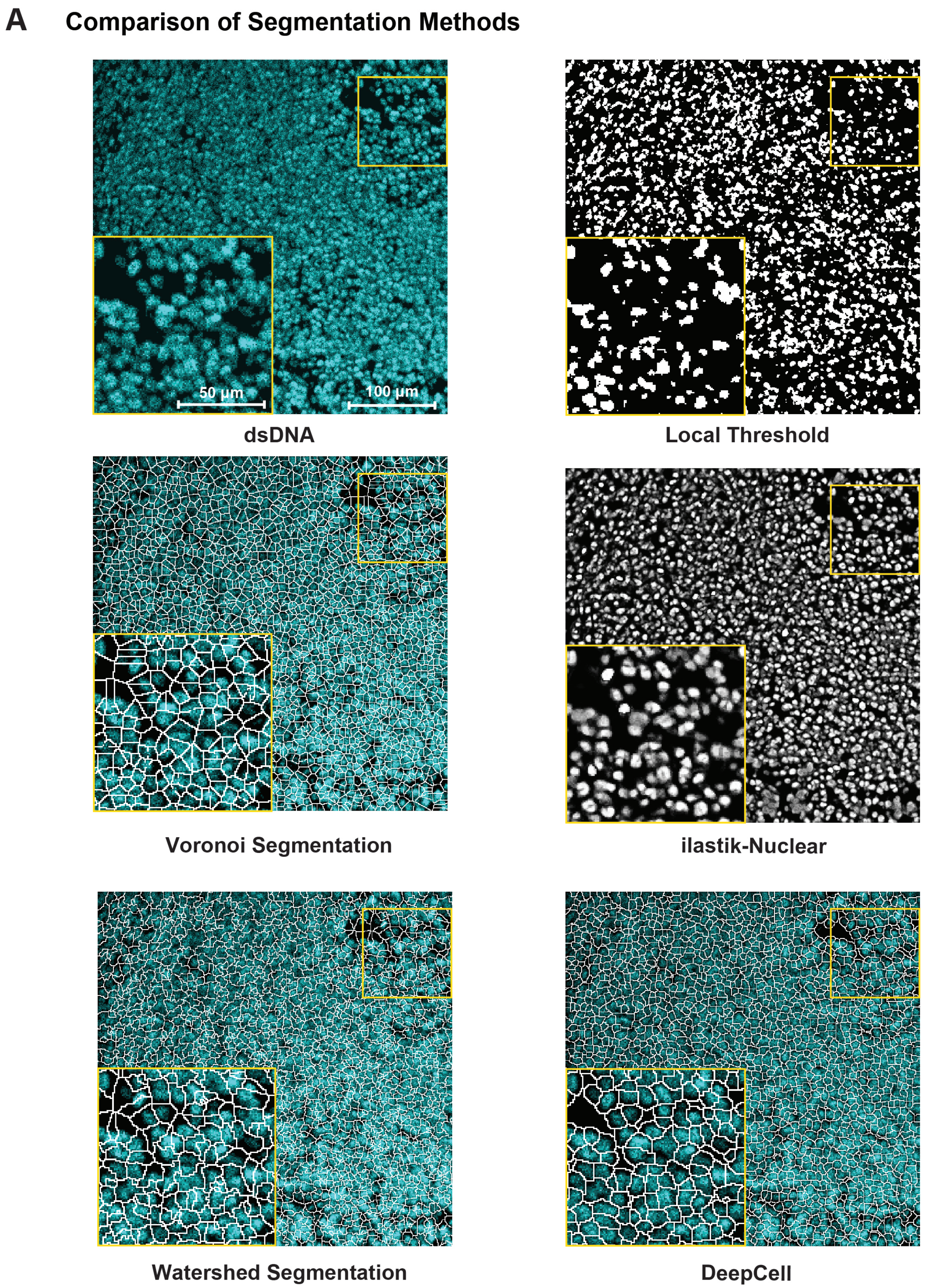

Figure S1: related to Figure 1. (A) A comparison of segmentation methods, on dsDNA images generated via MIBI, is shown here. The original dsDNA input in shown on the top left (cyan) and the various segmentation methods arranged there after.

B Examples of Cells with Membrane Markers Spillover into Adjacent Cells

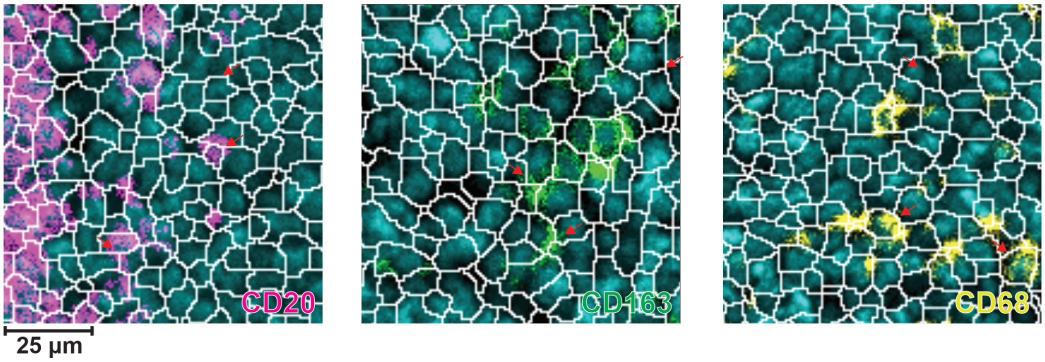

C User-defined Pixel and Shape Parameters for Border Signal Extraction

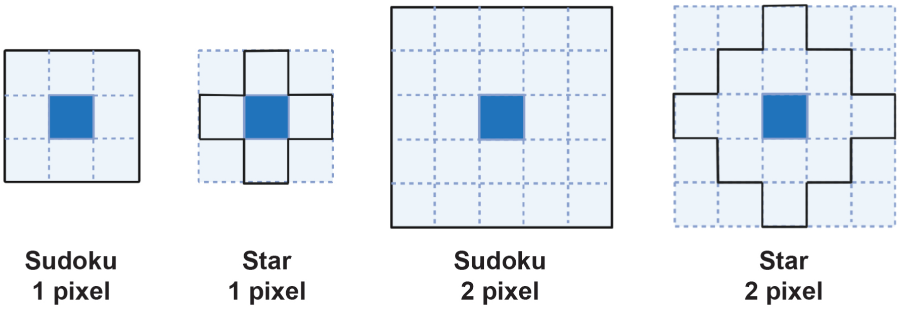

D Illustration of Individual Cell Scenarios for REDSEA Border Compensation

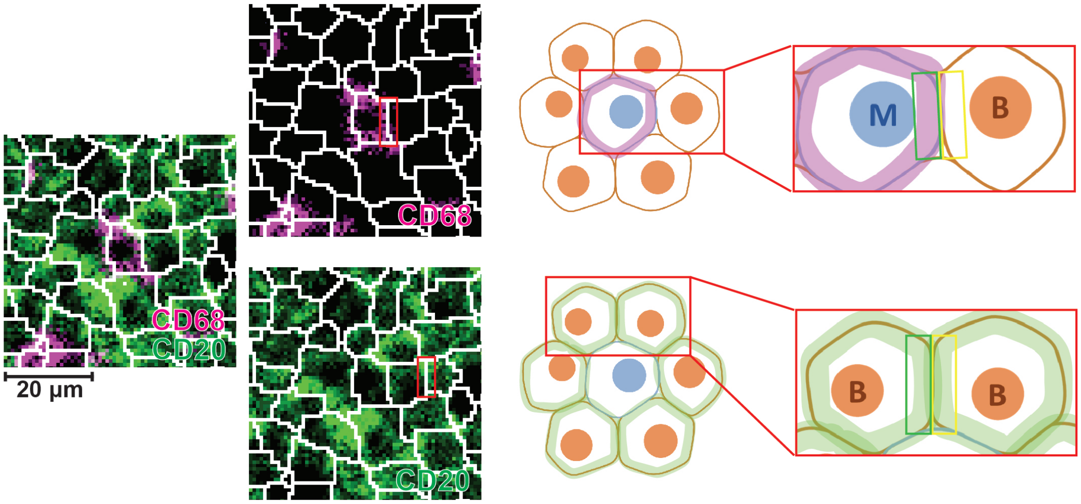

Figure S1 (cont.): related to Figure 1. (B) Examples of cells with cell-surface marker spillover into adjacent cells from well-segmented MIBI images. **(C)** Illustration of the user-defined pixel number and shape parameters for border signal extraction. **(D) Left:** A MIBI image focused on a macrophage ($CD68^{+}$, magenta) surrounded by B cells ($CD20^{+}$, green). Composite and single-channel images are shown. **Middle:** Cartoon representations of a macrophage (with a blue nucleus) surrounded by B cells (with orange nuclei) with only CD68 (magenta) or CD20 (green) signals shown. **Right:** Enlarged view of two different situations of signal spillover across cell boundaries encountered by the unsupervised REDSEA compensation: **Top:** A cell-pair with dissimilar surface-marker expression has asymmetric surface-marker distribution across the shared boundary (highlighted with green and yellow rectangles). For all pixel values extracted from the segmented CD68+ macrophage, REDSEA border algorithm first subtracts all selected counts across all markers present in the yellow rectangle, then adds all selected counts present in the green rectangle. The overall effect of REDSEA border compensation on this cartoon $CD68^{+}$ macrophage will be an increase of CD68 counts. Conversely, application of REDSEA border algorithm to the B cell adjacent to the macrophage results in a decrease of CD68 counts for the B cell (B), as signals from the green rectangle are subtracted and signals from the yellow box are added. **Bottom:** In a cell-pair with similar surface marker expression, the cells have roughly symmetric signals across their shared boundary (green and yellow rectangles), the REDSEA border compensation will result in a negligible change in compensated signals between similar cells.

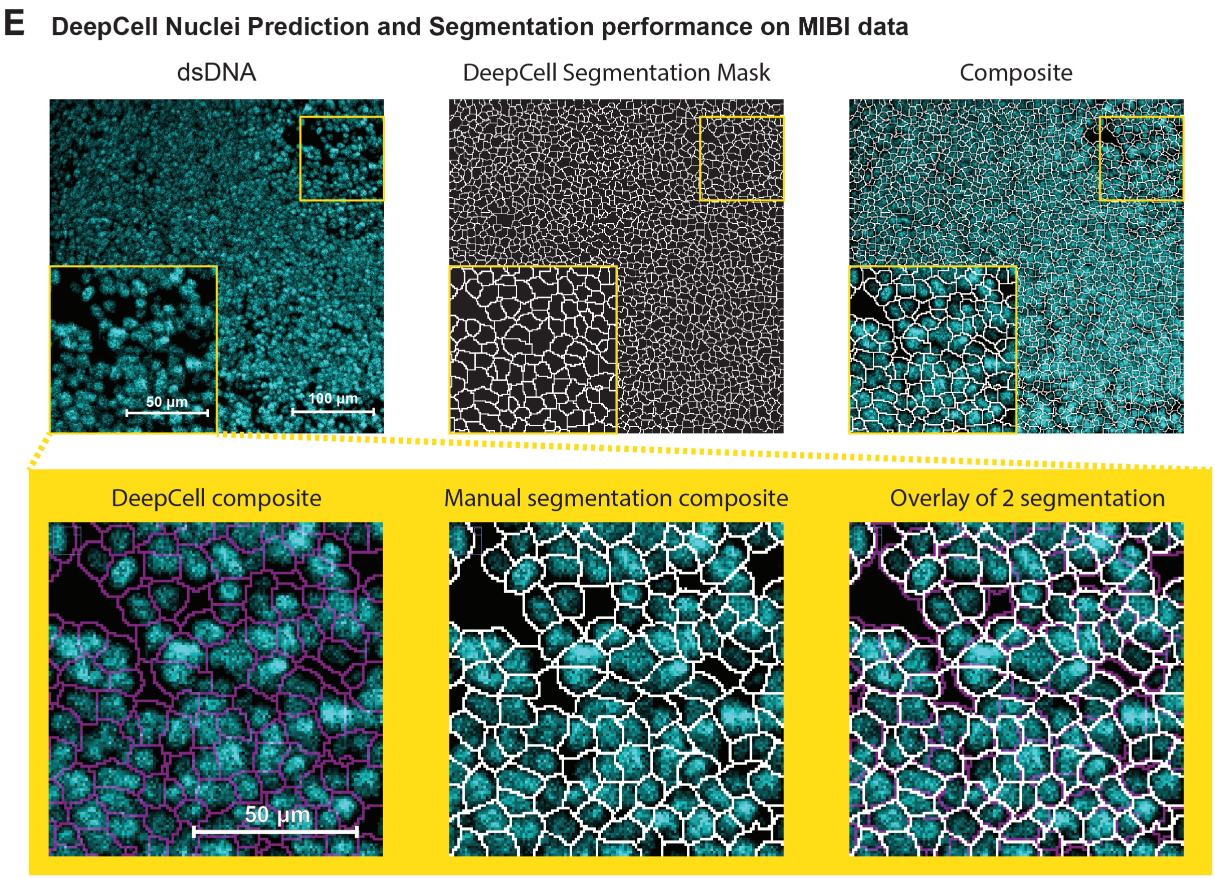

Figure S1 (cont.): related to Figure 1. (E) Top row: DeepCell segmentation of a single field of view using dsDNA (cyan). The region enclosed by the yellow box is enlarged in the bottom left of each image. Bottom row: A magnification of the yellow box from the top row is shown, with segmentation maps for DeepCell (left; magenta), manual segmentation (middle; white) and an overlay to compare the two segmentations (right; magenta and white). The dsDNA used for cell segmentation is shown in cyan.

F **CD4 and CD8 Double-Positive Cells Due to REDSEA Independent Artefacts**

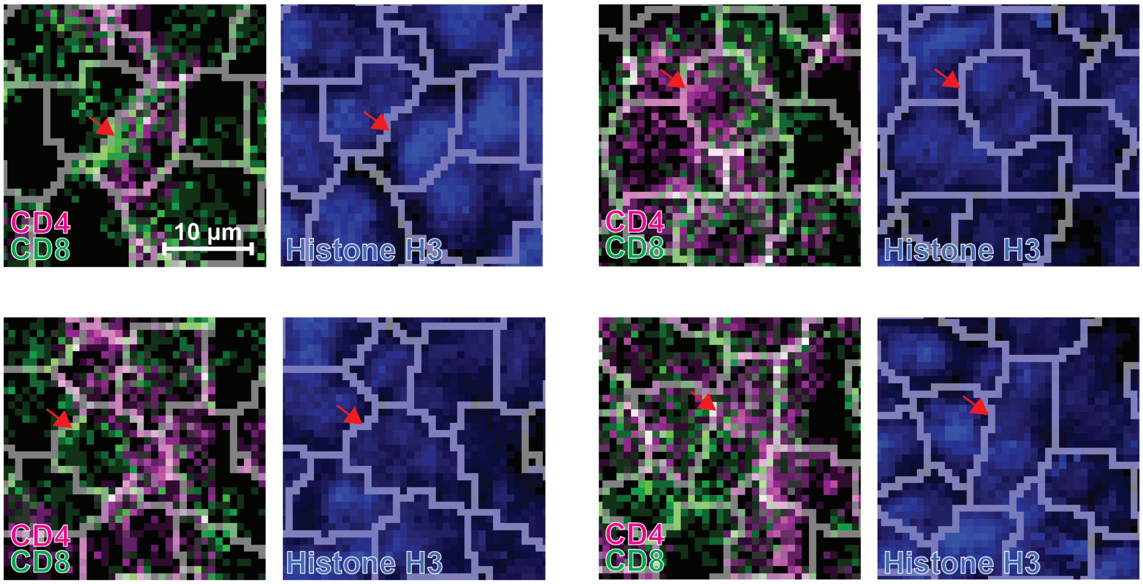

G **CD3 and CD20 Double-Positive Cells Due to REDSEA Independent Artefacts**

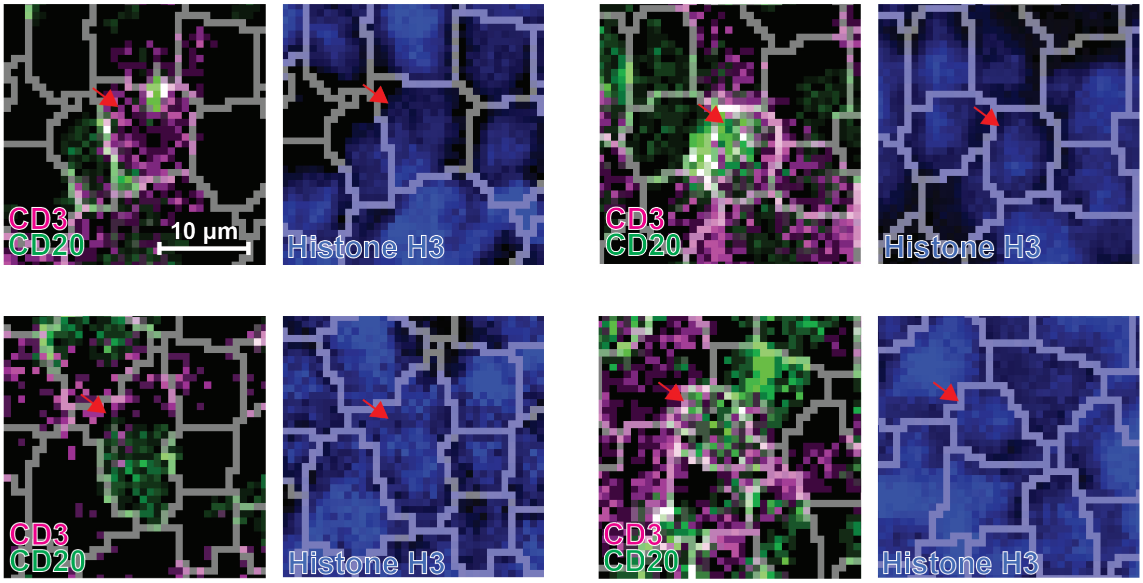

Figure S1 (cont.): related to Figure 1. (F) Representative images of CD4$^+$ CD8$^+$ double-positive cells still present after REDSEA border compensation, mainly due to poor segmentation or other REDSEA independent artifacts. **(G)** Representative images of CD3$^+$ CD20$^+$ double-positive cells still present after REDSEA border compensation, mainly due to poor segmentation or other REDSEA independent artifacts.
