# Supplementary material for: Adjacent Cell Marker Lateral Spillover Compensation and Reinforcement for Multiplexed Images
Source: Front Immunol. 2021 Jul 5;12:652631. doi: 10.3389/fimmu.2021.652631 (PMC8289709; doi:10.3389/fimmu.2021.652631)
Supplement: Supplementary file 2 [file DataSheet_2.pdf]

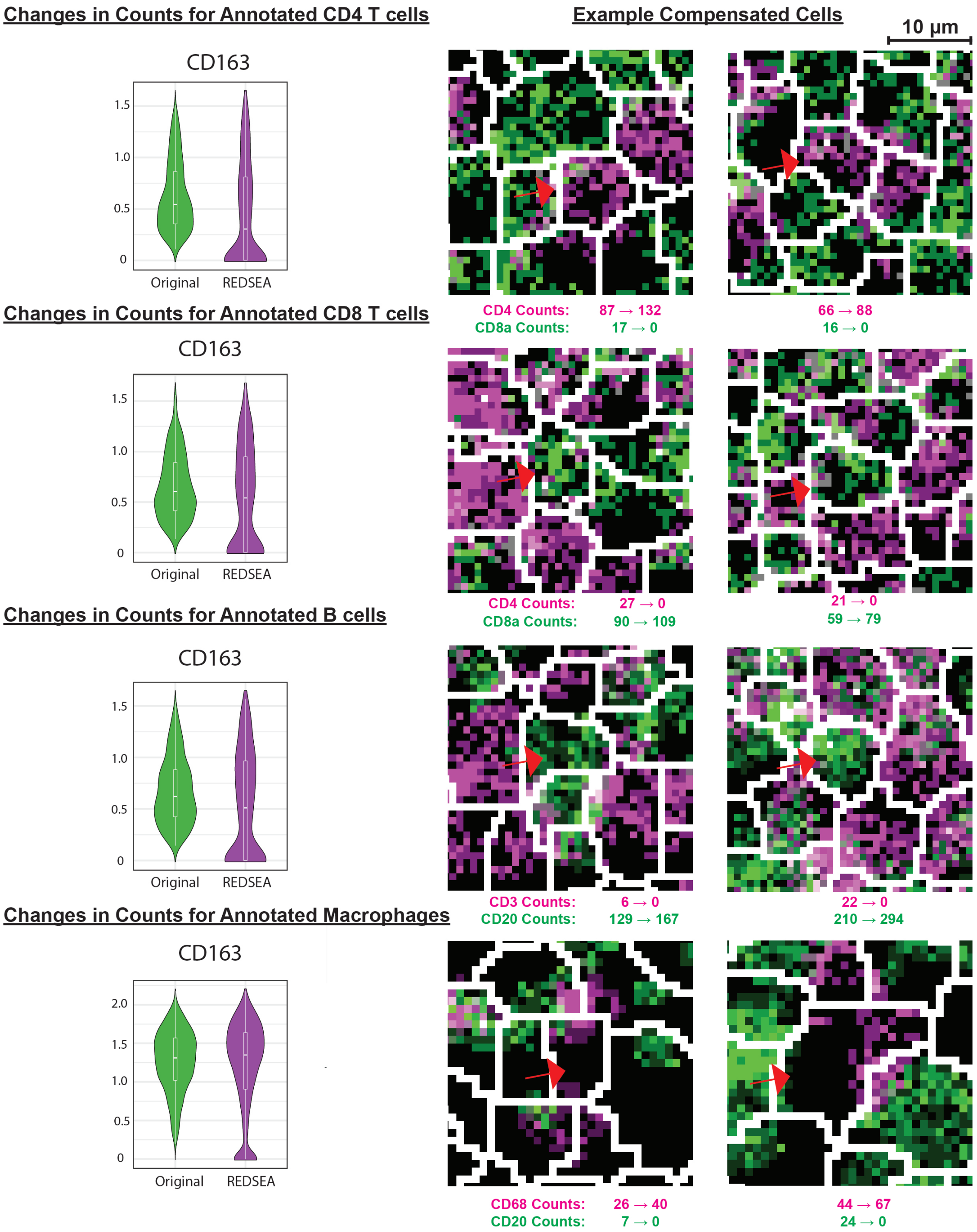

Figure S2: Related to Figure 2. Left: Transformed counts per cell for CD163 before and after REDSEA border compensation for each cell type identified. **Right:** Example images of each cell type and its marker counts before and after REDSEA compensation.
