# Supplementary material for: Adjacent Cell Marker Lateral Spillover Compensation and Reinforcement for Multiplexed Images
Source: Front Immunol. 2021 Jul 5;12:652631. doi: 10.3389/fimmu.2021.652631 (PMC8289709; doi:10.3389/fimmu.2021.652631)
Supplement: Supplementary file 3 [file DataSheet_3.pdf]

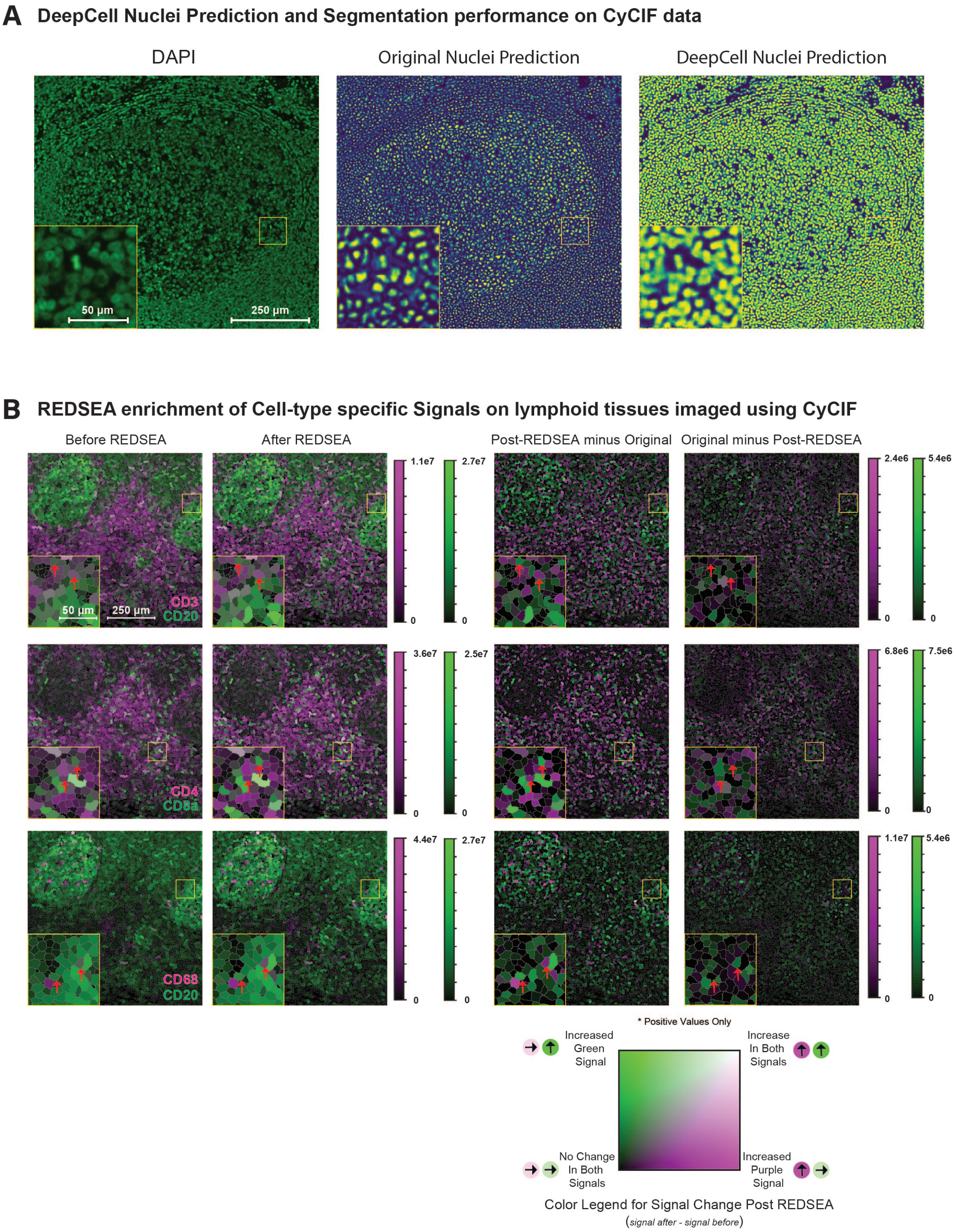


**Figure S3: Related to Figure 3. (A)** Left to Right: A representative CyCIF image of DAPI from human tonsil tissue (895 µm x 795 µm), the original provided ilastik nuclei prediction probability map, and the DeepCell prediction probability map generated for this study. **(B) Left:** A 900 µm x 900 µm CyCIF image of a human tonsil with color-scaled counts of the indicated markers within each cell before and after REDSEA compensation. The counts of three pairs of mutually exclusive markers are colored on the same scale for the segmented cells before and after compensation. **Right:** The signal difference of each cell from the image on the left shown before and after REDSEA compensation. Differences were calculated by subtracting the raw counts per cell from compensated counts or vice versa. Only positive values are colored as per the scale bar on the right. Red arrows indicate representative cells with successful REDSEA spillover correction.
