# Supplementary material for: Adjacent Cell Marker Lateral Spillover Compensation and Reinforcement for Multiplexed Images
Source: Front Immunol. 2021 Jul 5;12:652631. doi: 10.3389/fimmu.2021.652631 (PMC8289709; doi:10.3389/fimmu.2021.652631)
Supplement: Supplementary file 4 [file DataSheet_4.pdf]

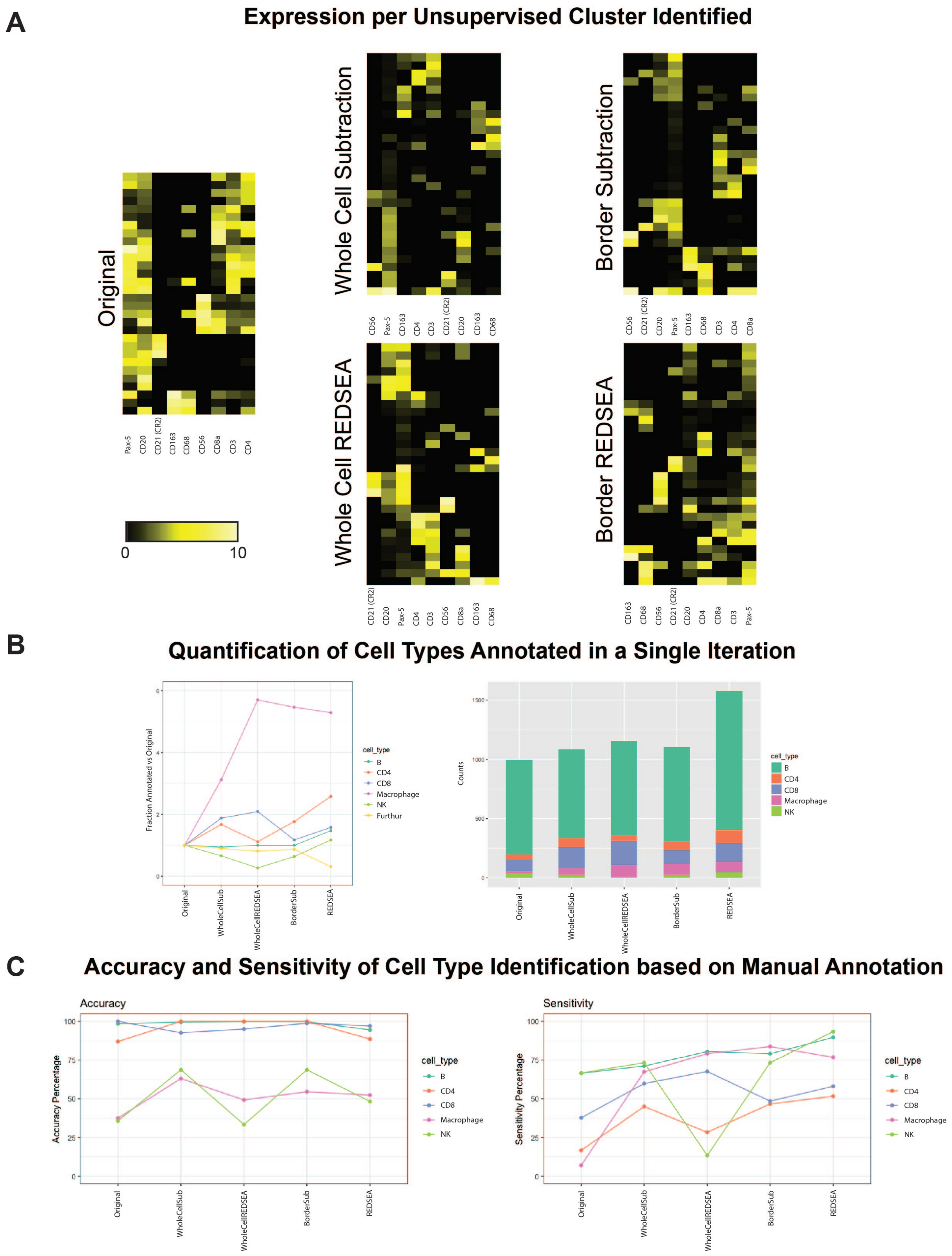

Figure S4: Related to Figure 4. (A) Marker Enrichment Modeling expression of each marker for each of the 30 clusters identified via FlowSOM, under the original (left) or each of the 4 compensation methods as denoted (middle and right). All clustering was performed independently for each condition, but with identical parameters. **(B)** Quantification of the number of cells for each cell type successfully annotated under each compensation condition, as represented by fold-change over cells annotated in the original condition (left) or as a cumulative plot (right). **(C)** The sensitivity (left) and accuracy (right) of cell-type identification under each compensation condition based on a manual consensus annotation from three independent individuals.

D

Median Marker Expression per Cell Type

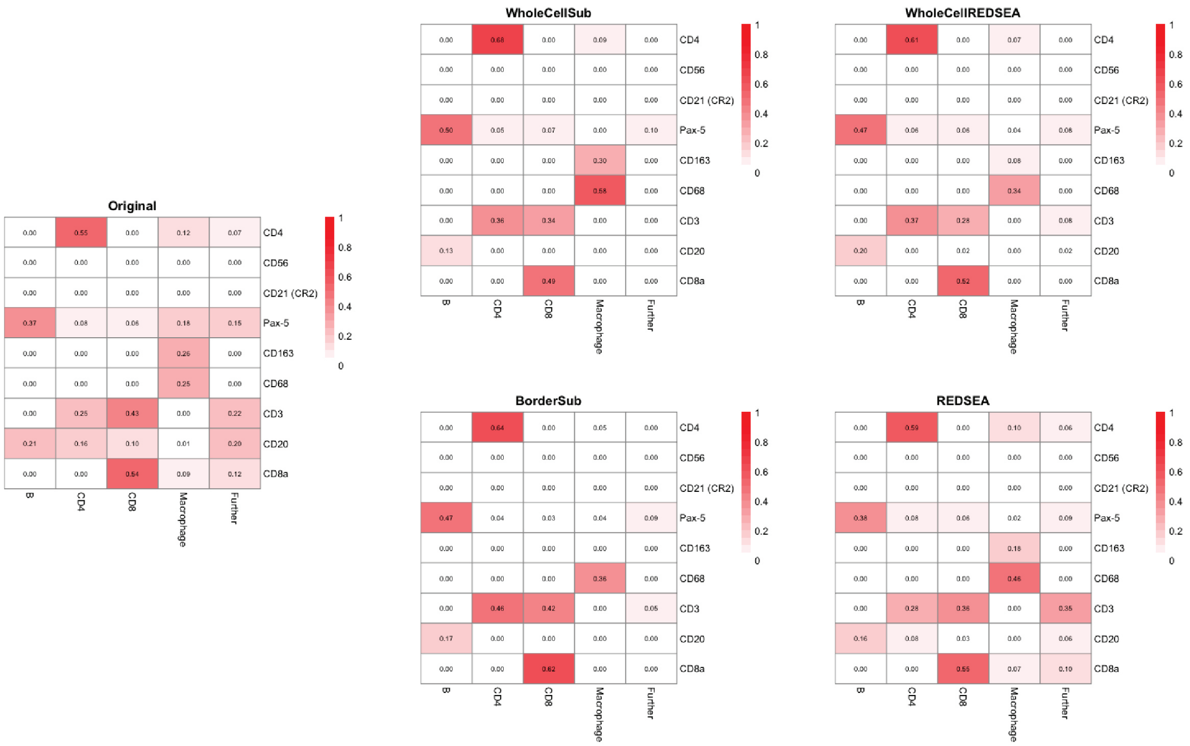

E

Phenotype Maps of Cell Types Identified

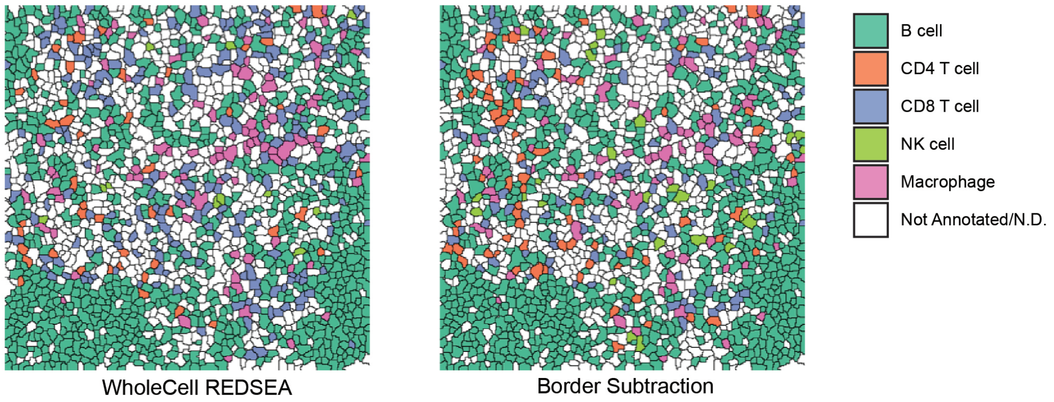

Figure S4 (cont.): Related to Figure 4. (D) The median marker expression of cell types identified under each condition colored according to a 0-1 (min-max) scale. **(E)** Phenotype maps obtained after Whole Cell REDSEA correction and Border Subtraction correction.
