# Supplementary material for: Adjacent Cell Marker Lateral Spillover Compensation and Reinforcement for Multiplexed Images
Source: Front Immunol. 2021 Jul 5;12:652631. doi: 10.3389/fimmu.2021.652631 (PMC8289709; doi:10.3389/fimmu.2021.652631)
Supplement: Supplementary file 5 [file DataSheet_5.pdf]

A Cells with Manual Annotation Errors that were Successfully Identified after REDSEA

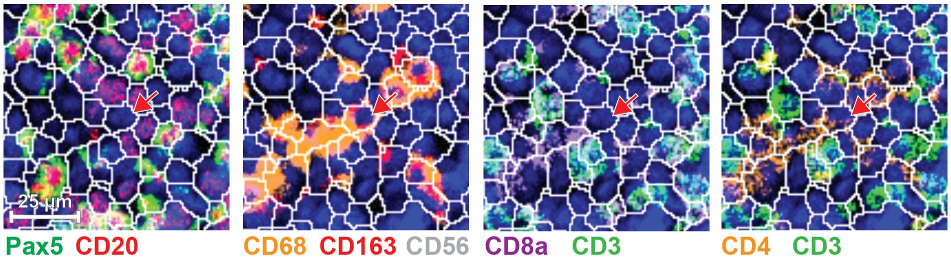

Original Result	Whole Cell Subtraction Result	Whole Cell REDSEA Result	Border Subtraction Result	REDSEA Result	Ground truth Number 1 Result	Ground truth Number 2 Result	Ground truth Number 3 Result	Ground truth Final Result
Not Determined	Macrophage	Macrophage	Macrophage	Macrophage	Not Determined	Macrophage	Macrophage	Macrophage

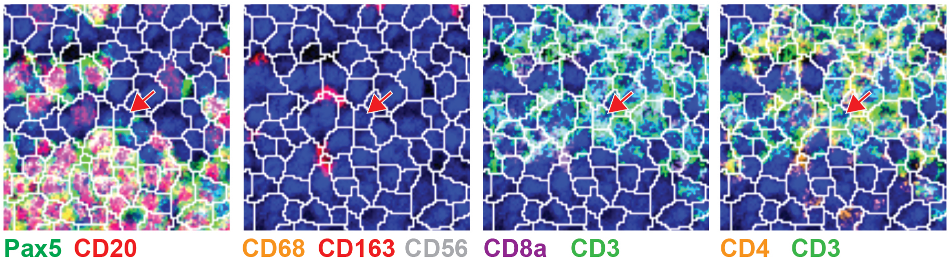

Original Result	Whole Cell Subtraction Result	Whole Cell REDSEA Result	Border Subtraction Result	REDSEA Result	Ground truth Number 1 Result	Ground truth Number 2 Result	Ground truth Number 3 Result	Ground truth Final Result
Not Determined	CD8 T	CD8 T	CD8 T	CD8 T	Not Determined	CD8 T	CD8 T	CD8 T

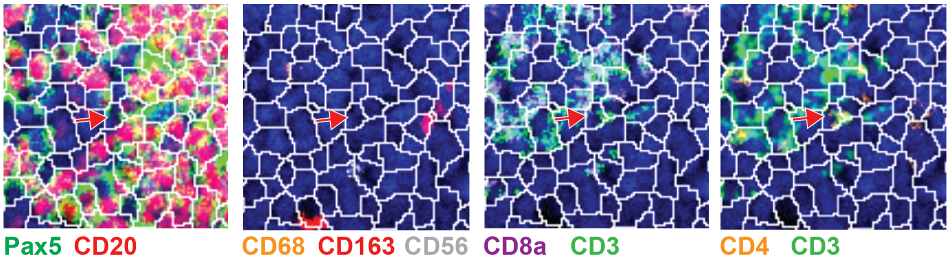

Original Result	Whole Cell Subtraction Result	Whole Cell REDSEA Result	Border Subtraction Result	REDSEA Result	Ground truth Number 1 Result	Ground truth Number 2 Result	Ground truth Number 3 Result	Ground truth Final Result
Not Determined	Not Determined	Not Determined	Not Determined	CD4 T	Not Determined	CD4 T	CD4 T	CD4 T

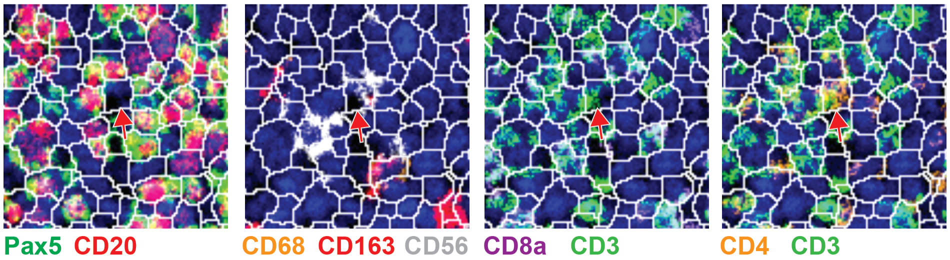

Original Result	Whole Cell Subtraction Result	Whole Cell REDSEA Result	Border Subtraction Result	REDSEA Result	Ground truth Number 1 Result	Ground truth Number 2 Result	Ground truth Number 3 Result	Ground truth Final Result
Not Determined	NK	Not Determined	NK	NK	Not Determined	NK	NK	NK

Figure S5: Related to Figure 4. (A) Representative images of cells with manual annotation errors (red arrows), but were successfully identified after REDSEA compensation.

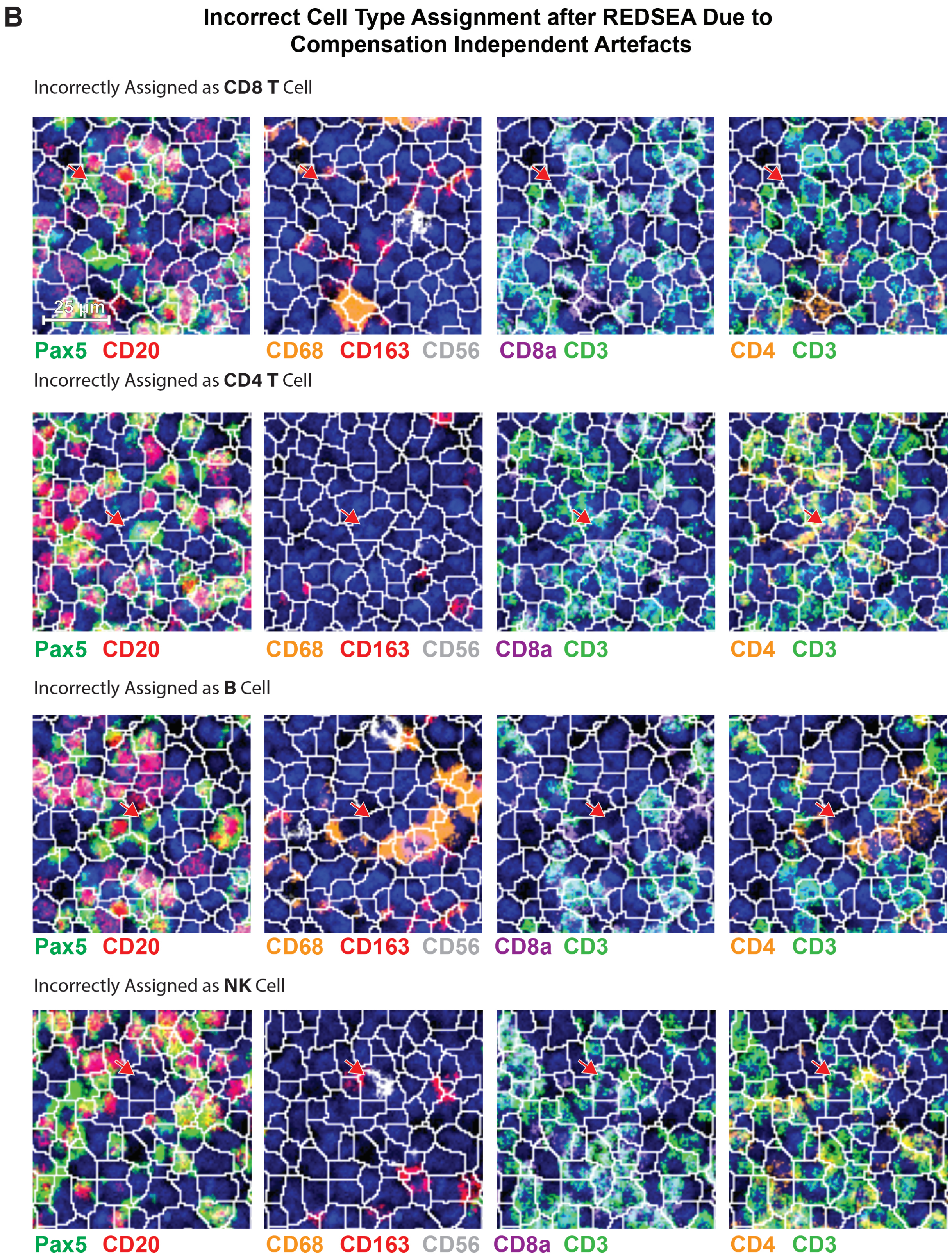

Figure S5 (cont.): Related to Figure 4. (B) Representative images of cells with incorrect cell type assignment after REDSEA (red arrows), due to compensation independent artifacts such as imperfect cell segmentation.
